# Trust in the public health system as a source of information on vaccination matters most when environments are supportive

**DOI:** 10.1016/j.vaccine.2022.06.012

**Published:** 2022-06-23

**Authors:** Sophie Lohmann, Dolores Albarracín

**Affiliations:** a Max Planck Institute for Demographic Research, Konrad-Zuse-Str. 1, 18057 Rostock, Germany; b Annenberg Public Policy Center, University of Pennsylvania, 202 S 36th St, Philadelphia, PA 19104, United States; c University of Illinois at Urbana-Champaign, 901 West Illinois Street, Urbana, IL 61801, United States

## Abstract

**Objectives:**

To understand whether health insurance coverage of vaccine costs and discussing vaccination with a healthcare provider are necessary for trust in CDC (Centers for Disease Control) to increase the uptake of the vaccine.

**Method:**

A nationally representative sample of 2,549 adults from the United States answered questions about trust in CDC, insurance coverage, interactions with healthcare providers, and risk perceptions, and then provided longitudinal reports of actual vaccination against influenza during the course of the 2018–19 flu season.

**Results:**

Trust in CDC as a source of information on vaccines was a strong precursor of vaccination. According to multilevel regressions, however, this effect was localized to respondents who had insurance coverage or whose providers discussed the vaccine with them. Further, the effect of trust was even stronger when both insurance coverage and healthcare provider discussions were present.

**Conclusions:**

Environmental factors supportive of vaccination increased the positive influence of trust in CDC on vaccine uptake by almost 50 percent. Insurance companies and healthcare providers can promote vaccination by covering the costs of vaccination and discussing vaccines in personalized conversations with patients.

## 1. Introduction

Although global health depends on vaccines [1], the individual and environmental determinants of vaccinations continue to be a puzzle: Even when vaccines against a disease are available, not everyone chooses receive the vaccine [2] and the world continues to witness protests against vaccine mandates [3,4]. In the United States, many scholars attribute reluctance to vaccinate to a lack of trust in health institutions, particularly the CDC (Centers for Disease Control). In addition, however, environmental factors appear to be paramount and may in many cases be a necessary condition for trust in health institutions to promote vaccination. Here, we examine the influence of institutional trust on influenza vaccination when cost is and is not covered by health insurance and when providers have and have not recommended the vaccine.

### 1.1. Trust in health institutions

Trust in the public health agencies that make vaccine recommendations is an important precursor of vaccination decisions and beliefs about vaccines [5–9]. Trust in vaccination is multidimensional and includes trust in the vaccine itself, in the provider who is administering the vaccine, and in the public health agencies that approve, issue recommendations, and provide information on the vaccinations [5]. Often, trusting the agencies that distribute this information is even more important than trusting the product itself—as a review of factors that contribute to vaccination-related attitudes concluded, “it is not *vaccines per se* that are mistrusted, rather it is the *institutions* (through which information about vaccines is delivered) that are mistrusted [emphasis in original]” [10](p7). In the United States, the primary body that recommends vaccines and communicates information about them are the Centers for Disease Control (CDC). Here, we therefore focus on trust in public health agencies (specifically, the CDC), a facet of trust that has been studied particularly often and found to be a crucial ingredient in decisions to vaccinate [5].

When people do choose to vaccinate, one of the most commonly cited reasons is that they received advice from trusted healthcare institutions such as national guidelines [10]. Conversely, when people do not vaccinate, a lack of trust in such institutions is a leading reason for their rejection of vaccines [8,9]. In the US, distrust in the CDC is fairly high (20–25% [11]) and worryingly, during disease outbreaks, trust in governmental information can decrease further as the situation in one’s own country worsens, leading to lower intentions to vaccinate [12–14]. This distrust matters because populations with lower trust in the public health system are about half as likely to be vaccinated (e.g., *OR* = 0.48) than populations with higher trust [15,16]. A literature review found that distrust in the government and its perceived motives for distributing health-related information is an important contributor to vaccine hesitancy across multiple countries [17], and an absence of trust in such information is one of the strongest predictors of vaccine misinformation [8]. In sum, prior research has shown that trust in public health institutions, such as the CDC, is a crucial factor in determining people’s attitudes towards vaccination.

### 1.2. Environmental factors

Trust, however, is only one factor in the vaccine puzzle and cannot lead to vaccination by itself. Instead, trust as an individual-level factor occurs in the context of environmental factors that can constrain or, conversely, facilitate vaccination. For example, people may trust the CDC’s information about vaccines but still not receive the vaccine because they cannot afford to or because they simply forget to do so. In this case, changing the environment around financial access or about reminders should allow these people to receive the vaccine. The real impact of trust may therefore be even greater than previously thought when considering environments supportive of vaccination.

In this research, we follow an ecosystem approach that highlights the multiple levels of influence on influenza vaccination behavior, from the individual to the community factors and, importantly, that these levels interact and therefore cannot be considered in isolation [18–21]. Ecosystem approaches recognize that the “conditions of life affect the health status and behaviors of individuals by constraining them” [22](p20). In other words, these approaches emphasize that individual-level characteristics such as trust operate in the context of broader factors, such as social networks, community characteristics, healthcare factors, and policies [21,23,24]. For example, community-based participatory research has shown how individual-level trust is deeply connected to environmental factors such as which healthcare services are available in a community, histories of racism or ableism, and insurance policies [25,26]. To illustrate, an individual’s trust in the vaccine’s safety may be important in determining whether these individuals vaccinate, but only when environmental factors allow them to vaccinate at all. In the most extreme case, when doses of the vaccine are not available in their country or there is no accessible vaccination site, people are physically constrained from vaccinating. Obviously, in this case they will not receive the vaccine no matter how positively they might feel about it or how much they might trust the health system. Here, we therefore examine the less obvious case of environmental factors that do not make vaccination impossible but merely more difficult.

A systematic review of vaccine hesitancy identified insufficient financial access and a lack of interactions with the healthcare system as the two primary environmental barriers within the midlevel ecosystem [27]. One-on-one interactions with healthcare providers who promote vaccines have been found to counteract a lack of trust in national institutions [5,28] and getting a personal recommendation from one’s primary care provider is frequently cited as one of the leading reasons for getting a flu shot [29]. Healthcare providers can use one-on-one interactions with their patients to contribute to vaccination in multiple ways: Providing personalized recommendations and answering questions [30], creating trust through relationship-building [31], and providing reminders to vaccinate [32]. These reminders are especially useful during a healthcare provider visit compared to, for example, receiving a reminder letter, because during a visit patients can immediately receive the vaccine without having to schedule and attend an appointment at a later time [33]. Furthermore, having insurance coverage for vaccines can act as a key facilitator [34,35]. The willingness to receive vaccines decreases with increasing cost [36,37] and even among healthcare workers, 33 percent indicate that they would not vaccinate if there were any out-of-pocket costs, no matter how low [38]. Depending on the disposable income that an individual has available, out-of-pocket costs can therefore make vaccination either prohibitively expensive or simply more burdensome, both of which can decrease vaccination rates. We thus studied the impact of trust in the CDC on adult vaccination in the context of personal discussions with providers and in the presence or absence of health insurance coverage of vaccines. A few prior studies have examined how the effect of trust depends on physical proximity to a disease outbreak [24] and on how much community-level trust exists [23], but to the best of our knowledge, how the effect of trust increases or decreases based on personal healthcare interactions and insurance coverage has not been ascertained. This work therefore contributes to the literature by not merely examining these factors side by side, but also by exploring the interaction between the individual level and the environmental level of the healthcare system.

To summarize, we tested a model in which vaccination behavior is determined by trust, which is moderated by environmental factors. This hypothesized pathway runs counter to a model in which vaccination behavior is instead determined mainly by risk perceptions, which implies that when the vaccine is recommended for somebody but they choose not to receive it, this is due to a mismatch between objective risk (as captured in the recommendation) and perceived risk (as captured in the person’s own evaluation). In these models, vaccine hesitancy is therefore ultimately based on a lack of accurate information about the disease and its risks [39–42]. Such models typically follow an expectancy-value approach and therefore describe three types of risk perceptions: The perceived likelihood of infection (in this case, how likely is it that I will get the flu this season?), the perceived severity of the disease (how dangerous is it to get the flu?), and the combined personal risk (i.e., the product of likelihood and severity). We therefore included these types of risk perceptions into our models as a well-established baseline against which to compare the effects of trust. Some reviewers of the vaccination literature have argued that the importance of risk perception has been overemphasized and that (mis)trust is a more important contributor to vaccine hesitancy [10,43], whereas others found that risk perceptions and trust matter equally [44]. Our analysis compares both risk perceptions and trust to better gauge the importance of trust, even after controlling for standard predictors of vaccination.

This paper therefore aimed to answer three research questions:

1. Is the association between trust and vaccination moderated by healthcare provider conversations and insurance coverage of the cost of the influenza vaccine?
2. How does the effect of trust on vaccination compare with that of risk perception in the presence of these environmental factors?
3. How does the effect of trust on vaccination compare with that of risk perception in the presence or absence of supportive structural environments?

We approached these questions using prospective data from a large, nationally representative sample. Importantly, this analysis extends ecosystem approaches to analyze the interplay of individual-level and environment-level factors rather than considering them as additive factors. This approach allows us to gauge how strong the true effect of trust on vaccination could be if important environmental constraints were removed from the healthcare system.

## 2. Methods

We analyzed a representative sample of adult Americans, who were part of a four-wave vaccination study conducted with the AmeriSpeak panel administered by NORC at the University of Chicago on behalf of the Annenberg Public Policy Center of the University of Pennsylvania during the 2018/2019 influenza season (September-March). AmeriSpeak is a probability-based panel that is representative of US adults with 97% coverage (for more details, see [45]). The sample used in this paper is a subset of a larger, multi-wave sample of vaccination. Data from this larger sample have also been used in previous papers [8,9,58–60], but these analyses have never been reported. Out of the 3,005 available participants, we used only the *N* = 2,549 for whom all variables of interest were available (the largest attrition came from the questions on insurance coverage of vaccines, which were missing for 425 respondents). Ninety-one percent of the surveys were administered online, the remaining participants were interviewed by telephone, and participants chose whether to take the survey in English or Spanish. The survey used a two-stage stratified sampling approach aiming for representativeness on gender and ethnicity among the adult population (18+; based on the February 2018 Current Population Survey). Survey weights were then used to account for the sample design and for non-response rates and all descriptive and inferential statistics were weighted to ensure representativeness in our analyses (for example, regression analyses were weighted and all reported means and percentages reflect weighted data). Participants’ ages ranged from 19 to 99, *M* = 48.17, *SD* = 18.01. Most participants were women (52%), non-Hispanic White (64%; 16% Hispanic, 12% Black, 4% Asian, 4% other/multiple), and college graduates (32%; 28% some college; 29% high school degree).

Participants were asked whether they had received the flu vaccine at any point during the flu season. Before the start of the study, 17 percent of respondents had already received their flu vaccine for the season and another 35 percent received the vaccine during the duration of the study. At the beginning of the study, trust in health officials was measured as “How much trust, if at all, do you have in [the CDC] to give you accurate information about the benefits and risks of vaccination?” (0 = *Very little trust at all*, 3 = *A great deal of trust*; M = 2.16, *SD* = 0.81). To assess financial access, participants indicated whether their insurance plan covered vaccinations, coded such that 1 = *Yes, with no co-pay (where the insurance company pays the whole cost)* (55%), 0.5 = *Yes, with co-pay (where you pay part of the cost) (30%), 0 = No or No, do not have health insurance at this time* (15%). Participants also indicated how often they had discussed the flu vaccine with “your doctor or some other medical professional” in the past year (0 = *Never*, 34%; 1 = *Once*, 48%; 2 = *More than once*, 18%).

Two variables were used to assess risk perception: Perceived likelihood of infection was measured with the item “Just your best guess, how likely, if at all, do you think you are to get infected with the flu this flu season?” (1 = *not likely at all* to 4 = *very likely*; ; *M* = 2.17, *SD* = 0.75)^1^. Perceived severity was measured with the item “Just your best guess, how severe (e.g., life threatening, causing major illness), if at all, do you think complications from the flu can be?” (1 = *not severe at all* to 4 = *very severe*; *M* = 3.08, *SD* = 0.87).

Importantly, we assessed the importance of environmental healthcare system factors above and beyond standard indicators of individual-level socio-economic status: We controlled for education (measured in four categories: no high school degree, high school degree, some college, college degree or higher) and income (coded into 8 categories ranging from $0 – 5,000 to > $150,000). Further, we controlled for race and ethnicity (collapsed into five categories: Hispanic, non-Hispanic Asian, non-Hispanic Black, non-Hispanic White, and non-Hispanic other race), gender (measured as either male or female), and age.

To predict the likelihood of vaccination, we used a random-slopes multilevel binomial model in which respondents were nested within the states they resided in. The predictors of interest were trust in the CDC’s information, whether respondents had discussed the flu vaccine with healthcare providers, and whether their health insurance covered vaccines. All possible interactions between these three variables were included into the model, alongside additive effects of the demographic control variables. The two risk perception variables were also included in the model, along with their two-way interaction. To allow easier interpretation of regression coefficients, we z-standardized the variables that did not have a zero point (i.e., age, perceived likelihood of infection, and perceived severity). The outcome variable was a binary indicator of whether respondents had received the vaccine by the end of the flu season (1 = *Yes*, 0 = *No*).

## 3. Results

First, we discuss the lower-order effects that emerged from our multi-level binomial random-slopes model. Then, we show how the three-way interaction between trust in the CDC, discussions with healthcare providers, and insurance coverage of the vaccine qualified these lower-order results. The detailed regression results appear in Table 1. The data confirmed that all three predictors of interest were important determinants of vaccination behavior: Even in the absence of other factors, discussing the vaccine with a healthcare provider was associated with sevenfold increased odds of getting vaccinated for the average person, *OR* = 7.02, 95 % CI [2.45, 20.05]. Similarly, even without healthcare provider discussions and with low trust, having full financial coverage through one’s insurance was associated with almost sixfold higher odds of vaccinating, *OR* = 5.94 [1.23, 28.75]. The odds of vaccinating were also 75% higher when participants reported one point higher trust in the CDC (on a 4-point scale), *OR* = 1.73 [1.17, 2.56]. Two of the demographic controls were significant, showing that being Hispanic rather than non-Hispanic White, *OR* = 1.35 [1.02, 1.78], and being one standard deviation (~18 years) older, *OR* = 1.27 [1.15, 1.40], were both associated with higher vaccination rates.

Our model further allowed us to evaluate the influence of risk perceptions on influenza vaccination in adults. We used three different operationalizations of risk perception: perceived severity, perceived likelihood of infection, and their product. Contrary to expectations, none of these types of risk perception showed any appreciable association with vaccination behavior when controlling for the other variables in our model, perceived severity *OR* = 1.02 [0.93, 1.12], perceived likelihood *OR* = 0.98 [0.89, 1.07], interaction *OR* = 1.03 [0.95, 1.13]. To test whether this absence of association could be explained by the other variables included in our model (e.g., mutual suppression effects between risk perception and trust), we ran another exploratory model with only the two risk perception variables, their interaction, and the demographic controls. Again, however, risk perceptions did not emerge as significant predictors of vaccination; perceived severity *OR* = 1.07 [0.98, 1.17], perceived likelihood *OR* = 0.97 [0.89, 1.06], interaction *OR* = 1.06 [0.98, 1.15].

**Fig. 1.**
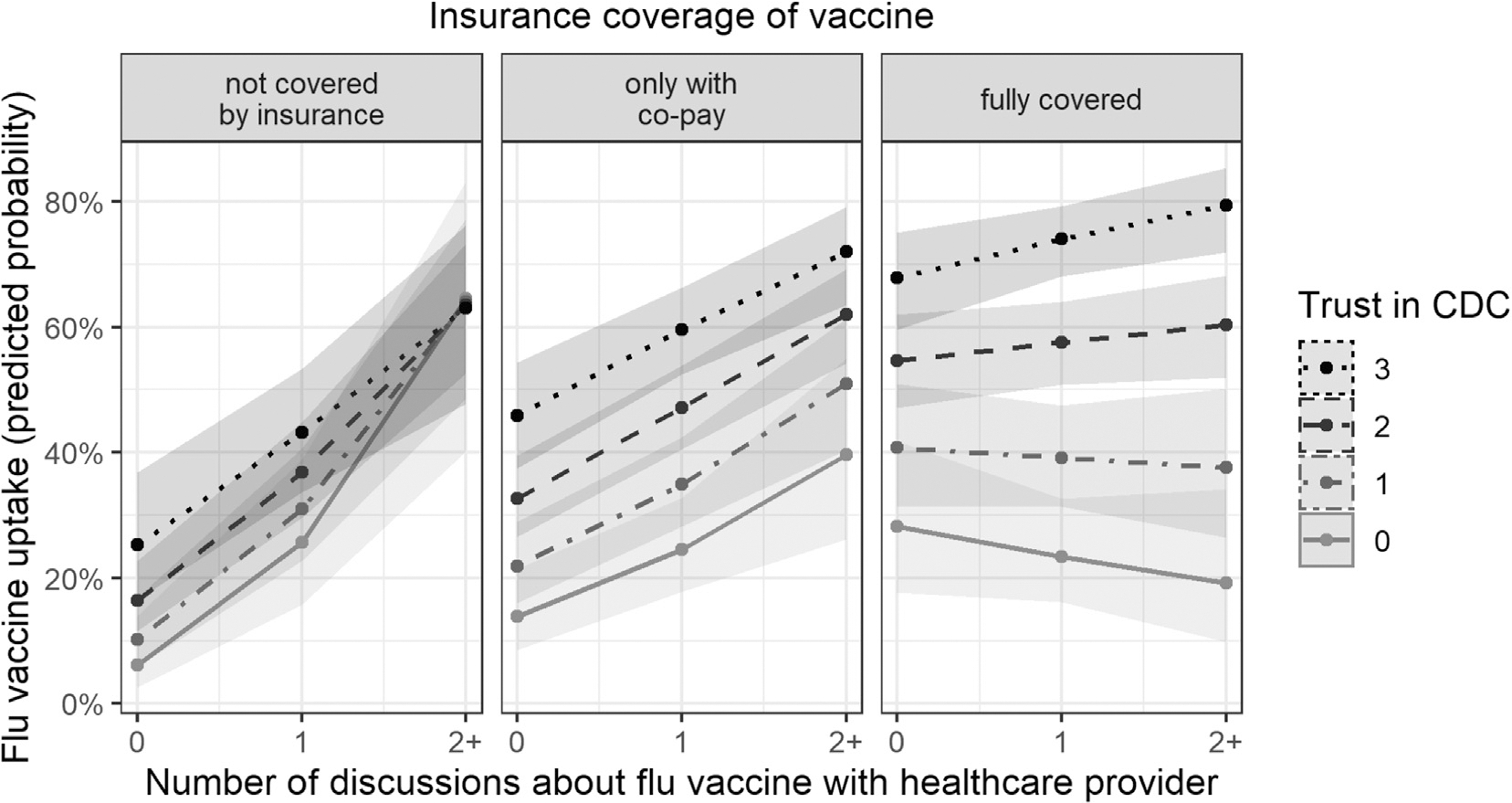
Predicted probabilities (marginal effects) of receiving the flu vaccine among a representative US population (2018–19 flu season) as a function of financial access, discussions with healthcare providers, and trust in the CDC as a source of information about vaccination.

Next, we review the two interactions that emerged as significant from our main model. First, a significant two-way interaction between discussions with healthcare providers and insurance coverage, *OR* = 0.15 [0.06, 0.39] showed that vaccination rates were particularly high when both healthcare providers and insurance providers actively supported vaccination (through discussions and financial coverage, respectively). Second, this two-way interaction and the main effects were qualified by a three-way interaction between discussions with healthcare providers, insurance coverage, and trust, *OR* = 1.60 [1.04, 2.47], plotted in Fig. 1.^2^

This interaction showed that trust mattered most when environments were supportive. In particular, trust exerted the strongest effect on vaccination when healthcare providers gave reminders in personal discussions and when the insurance completely covered the vaccine. For example, higher trust increased the likelihood of vaccination only slightly when respondents had neither discussions about nor financial coverage of vaccination (from 6% to 25%; the effect of trust was *OR* = 1.73 here). In contrast, higher trust increased the likelihood of vaccination much more when respondents had multiple discussions and insurance coverage of all of the costs of vaccinations (from 19% at low trust to 79% at high trust; the effect of trust thus increased to *OR* = 2.53). Furthermore, supporting environmental factors partially counteracted the effect of having moderate (but not low) trust: Respondents with moderate trust who talked to healthcare providers or whose insurance paid all costs were more likely to vaccinate than those with higher trust but no such supporting factors.^3^

We conducted additional analyses to explore the unexpected positive coefficient for Hispanic compared to non-Hispanic White participants. Without any other controls in the model, Hispanic participants showed non-significantly lower vaccination rates than non-Hispanic White participants, *OR* = 0.83 [0.66, 1.06]. This coefficient flipped once we included age, trust in the CDC, and insurance status into the model: Because Hispanic participants on average were younger, reported lower trust, and were less likely to have insurance coverage, the results changed after accounting for these three factors, *OR* = 1.33 [1.02, 1.74], suggesting suppression effects which we discuss below.

## 4. Discussion

Our results showed first that trust in the CDC as a source of vaccine information mattered when environments supported vaccination, particularly when multiple supporting factors were in place. The presence of at least one supporting factor (personal reminders or financial access) was also effective at increasing vaccination among people with only medium trust. However, environmental facilitators mattered much less for people with low trust, who remained unlikely to be vaccinated regardless of conversations or insurance coverage. Trust therefore emerged not as a sufficient, but as a necessary predictor: In the absence of trust, vaccination was highly unlikely, no matter how favorable other factors were. Our research question (1) *Is the association between trust and vaccination constrained by lack of financial access and conversations with healthcare providers?* can therefore be answered affirmatively. We found that the effect of trust increased from *OR* = 1.73 to *OR* = 2.53 between people faced with environmental constraints versus environmental facilitators, even after controlling for demographic predictors. The strength of this association that we find in non-supportive environments is similar to what previous analyses have reported as the overall effect size for the association [15,16,46,47]. Crucially, however, our analysis is the first to show that the strength of this association increases by almost 50% when considering environments that are supportive of vaccination. This finding thus suggests that actors in the healthcare system can make important contributions by reducing constraints on vaccination, allowing the public health system to reap the benefits of trust, which otherwise remain underrealized.

At the same time, however, risk perceptions about influenza were not associated with vaccination rates in our sample. Our research questions (2) and (3) asked how the effect of trust on vaccination compares to that of risk perception in the presence or absence of structural constraints, respectively. We found that trust in the CDC was markedly more strongly associated with seasonal influenza vaccination than were risk perceptions, even when vaccination behavior was constrained by lack of financial access and lack of personal interactions with healthcare providers. This difference increased even more in supportive structural environments as the effect of trust on vaccination grew stronger. The null effect for risk perception ran counter to our expectations and counter to the literature. For example, a meta-analysis of 34 studies found that perceived severity (meta-analytic *r* = 0.16) and perceived likelihood of infection (meta-analytic *r* = 0.26) were both significantly associated with vaccination behavior, whereas the equivalent correlations in our data were *rs* = 0.05 and −0.04. Possible reasons for this discrepancy include our sample composition which was representative of the US population, and therefore comprised largely of healthy people. Further, our questions asked about unconditioned risk, namely the perceived overall likelihood of getting the flu, rather than about conditioned risk, namely the perceived likelihood of getting the flu if one does not receive the vaccine. Conditioned risk questions tend to produce larger effects [39]. However, the question on perceived severity should be unaffected by these differences and still resulted in a null effect. It is noteworthy that our sample is both representative and larger than any single study included in the most recent meta-analysis of risk perceptions on influenza vaccination [39]. Prior studies may have overestimated the effect of risk perception due to underpowered analyses and other sample biases.

Further, it is worth noting that our results showed a higher vaccination rate among Hispanic participants than among non-Hispanic participants. This result was unexpected given prior work that found vaccination disparities such that Hispanic people and other People of Color are often vaccinated at lower rates than non-Hispanic White people [48,49]. Through supplementary analyses, we found that this counterintuitive result was influenced by suppression effects of age, trust, and insurance status. When ethnicity was the only predictor of vaccination, we found no difference in vaccination status. However, because higher age, higher trust, and insurance coverage were all strong predictors of vaccination and Hispanic participants on average reported lower age, lower trust, and lower insurance coverage, we observed higher average vaccination rates after accounting for these differences.

Our results are based on a nationally representative sample but carry limitations. First, all data were self-reported. One third of respondents in our survey said that their insurance either did not cover flu shots or required a co-pay. Under the Affordable Care Act, however, insurance plans must cover the cost of flu shots without charging a co-pay. Some of these respondents were likely enrolled in grandfathered plans, which were established before the Affordable Care Act and are not bound by the same rules, but outside sources estimated that such plans applied to only 13% of workers at the time of the survey [50]. Grandfathered plans therefore cannot explain the size of the discrepancy. Our data capture only subjective costs of vaccination, which can differ from the objective costs if people are mistaken about what their insurance covers. An alternative reading of our results on environmental influences is therefore that health insurance plans need to better communicate their cost structures and the benefits to which people are entitled. Second, our data cannot say how the results would change in at-risk populations with chronic health conditions. The closest approximation in our data was age, as older adults are known to be at higher risk of complications from influenza [51]. Our results held when controlling for age, but future studies should attempt to replicate the structural constraints on trust that we presented in at-risk groups such as pregnant women or adults with cardiovascular disease. Third, our analyses examined only trust in the US Centers for Disease Control. The CDC represents one of the best-known and most visible healthcare actors on the national level [11], which is why trust in the CDC is a common proxy for trust in the national healthcare system. Nevertheless, trust is a multi-dimensional concept and trust in other healthcare institutions may play a role in decisions to vaccinate as well. Future research should therefore examine how other forms of trust, for example in the FDA vaccine approval process, pharmaceutical companies, and state or county health departments operate to predict vaccination behavior within environmental constraints. Fourth, similar to how trust is a multidimensional concept, it is important to note that vaccinations against infectious diseases involve risk perceptions along multiple dimensions, such as the likelihood and severity of infecting someone else with the disease [52,53], the likelihood and severity of adverse side effects from the vaccine [54,55], and the likelihood and severity of contracting the disease [39,42]. When risk of infection and risk of the vaccine were directly compared in expectancy-value studies of pandemic influenza and seasonal influenza, both contributed to the intention to vaccinate and ultimately to vaccination behavior, but the effect of perceived risk of infection was stronger [54,55]. In the present study, we focus only on the perceived risk from getting infected with the flu.

Fifth, we analyzed the role of trust in only one adult-age vaccine and future research should examine whether the results extend to other vaccines. If these results generalize, the insights on trust presented here hold important implications for other new or emerging adult-age vaccines against viruses such as SARS-CoV-2, malaria and HIV. Knowledge about who will be willing to vaccinate is therefore crucial for forecasting public health and developing interventions as more vaccines targeted at adults become available. This research extends the literature on ecosystem approaches in vaccination [20,21] by applying these theories to the domain of trust and providing a concrete example of how interactions between levels of influence can alter the effect of any one variable. In this context, it is also important to highlight that the results included an interaction between two environmental factors: Discussions with healthcare providers were particularly effective when vaccination was covered and insurance coverage was particularly effective when healthcare providers discussed the vaccine with patients. Our results therefore suggest a complex interplay of factors in which attention needs to be paid both to interactions between levels and interactions within levels. To be effective at increasing vaccination behavior, interventions aimed at individual-level characteristics such as increasing trust [56,57] may need to be immersed within an environmental context that supports vaccination.

In conclusion, this study showed that trust in the national health system is important in the decision to receive the seasonal influenza vaccine, but that its effect depends on structural factors. A lack of financial access and one-on-one conversations with healthcare providers prevent vaccination regardless of the level of trust in CDC people report. Conversely, when vaccination is facilitated by full-coverage insurance policies and repeated conversations with healthcare providers, the effect of trust increases by almost 50%. In contrast, risk perceptions were not connected to vaccination rates in this nationally representative sample of mostly healthy adults. To reduce constraints on vaccination behavior, insurance coverage or free vaccination as well as repeated encouragement from healthcare providers are needed to ensure high coverage levels and ultimately herd immunity.

## Figures and Tables

**Table 1:** Regression coefficients of multi-level binomial regression (outcome: flu vaccination).

Term	OR	95 % CI

(Intercept)	0.06	[0.03, 0.16] *
Discussions with healthcare providers	5.27	[2.53, 10.99] *
Insurance coverage	6.01	[1.99, 18.11] *
Trust in CDC	1.73	[1.17, 2.56] *
Perceived severity (z-standardized)	1.02	[0.93, 1.12]
Perceived likelihood of infection (z-standardized)	0.98	[0.89, 1.07]
Education: no high school degree	1.13	[0.80, 1.61]
high school degree	0.81	[0.63, 1.05]
some college	0.92	[0.72, 1.19]
college degree (reference group)	–	–
Income: < 5 k	1.11	[0.59, 2.10]
5–10 k	0.67	[0.37, 1.23]
10–20 k	1.05	[0.73, 1.49]
20–40 k	1.16	[0.87, 1.54]
40–60 k	1.11	[0.84, 1.48]
60–100 k (reference group)	–	–
100–150 k	1.05	[0.77, 1.43]
150 + k	1.00	[0.70, 1.42]
Race/Ethnicity: Hispanic	1.35	[1.02, 1.78] *
non-Hispanic Asian	1.11	[0.66, 1.89]
non-Hispanic Black	0.96	[0.70, 1.32]
non-Hispanic other race	0.70	[0.42, 1.17]
non-Hispanic White (reference group)	–	–
Gender: male	1.08	[0.90, 1.30]
female (reference group)	–	–
Age (z-standardized)	1.27	[1.15, 1.40] *
Discussions with HCPs × Insurance coverage	0.15	[0.06, 0.39] *
Discussions with HCPs × Trust in CDC	0.75	[0.54, 1.05]
Insurance coverage × Trust in CDC	1.01	[0.62, 1.66]
Perceived severity × Perceived infection likelihood	1.03	[0.95, 1.13]
Discussions with HCPs × Insurance coverage × Trust in CDC	1.60	[1.04, 2.47] *

CDC = Centers of Disease Control. HCPs = Health Care Providers

## Footnotes

1 The 9 participants who had already had the flu at this point were excluded from this question.

2 We also examined a model in which participants whose insurance did not offer any co-pay and participants without any insurance were separated instead of collapsing them into the same category. Descriptively, this model yielded a similar pattern of results, main effect of insurance coverage *OR* = 7.46 [2.11, 26.40], 2-way interaction between healthcare provider discussions and insurance coverage *OR* = 0.12 [0.04, 0.37], 3-way interaction *OR* = 1.70 [1.04, 2.78]. However, the number of participants who reported having an insurance but not receiving any co-pay was very low, *n* = 60, resulting in group sizes of <20 0 in the interactions. The main text therefore presents only the model with both categories collapsed to increase the reliability of our results. Similarly, we did not include additional interactions with the indicators of risk perception to reduce model complexity and because group sizes would have become too small and would have affected statistical power. When we conducted exploratory robustness checks controlling for interactions between risk perception and the other predictors, power was consequently so low that almost no effects were significant. However, the 3-way interaction presented in the main text remained similar in size, *ORs* = 1.51 and 1.57, compared to *OR* = 1.60).

3 In addition to these results, the graph may suggest that when multiple discussions had taken place and trust was low, higher insurance coverage counterintuitively seemed to imply lower chances of vaccination. However, this combination of variables was rare in the sample, so that these estimates were based on <10 people per group (in contrast to an average sample size of 83.43 in each of the other groups) and should therefore be interpreted with caution.
